# The Impact of C-Peptide and Diabetes Mellitus on Coronary Ectasia and Effect of Coronary Ectasia and C-Peptide on Long-Term Outcomes: A Retrospective Cohort Study

**DOI:** 10.1155/2022/7910566

**Published:** 2022-10-08

**Authors:** Kenan Toprak, Mustafa Kaplangoray, Ali Palice

**Affiliations:** ^1^Department of Cardiology, Faculty of Medicine, Harran University, Sanliurfa, Turkey; ^2^Mehmet Akif İnan Training and Research Hospital, Sanliurfa, Turkey

## Abstract

**Background:**

Coronary artery ectasia (CAE) is an entity frequently associated with atherosclerotic coronary artery disease (CAD) in clinical practice. Although it has common risk factors with atherosclerotic CAD in its development, the pathophysiology of CAE is not fully known and it is not seen in every CAD suggesting that different determinants may play a pivotal role in the development of CAD. This study aimed to reveal the impact of C-peptide and diabetes mellitus (DM) on CAE and the effect of C-peptide and coronary ectasia on long-term outcomes in patients who underwent coronary angiography.

**Methods:**

A total of 6611 patients who underwent coronary angiography were followed up retrospectively, and their major adverse cardiovascular event (MACE) status of an average of sixty months was recorded. According to their angiographic features, the patients were divided into two groups those with and without CAE. MACE development was accepted as the primary endpoint.

**Results:**

A total of 552 patients had CAE and MACE developed in 573 patients. Patients with CAE and higher C-peptide levels (Q4 + Q3) showed higher rates of MACE as compared to those without CAE and lower C-peptide levels (Q1 + Q2) (20.8% vs 7.6%; 70.1% vs 29.1%; *p* < 0.001, for both of them). In multivariate regression analysis, high C-peptide levels were determined as an independent risk factor for CAE (OR 2.417; 95% CI 2.212–2.641; *p* < 0.001). The Kaplan–Meier cumulative survival curves showed that the risks for MACE increased as the C-peptide levels increased. The Cox regression analysis for 5-years MACE related to the plasma C-peptide levels and presence of CAE, C-peptide, and CAE were found to be independent predictors of MACE (HR = 1.255, 95% CI: 1.164–1.336, *p* < 0.001 and HR = 1.012, 95% CI: 1.002–1.023, *p*=0.026, respectively).

**Conclusion:**

Our study revealed that a high C-peptide level is an independent risk factor for CAE and that CAE and C-peptide are independent predictors for the development of MACE.

## 1. Introduction

Coronary artery ectasia (CAE) is a type of coronary artery disease (CAD) that is defined as the enlargement of a coronary artery segment to at least 1.5 times the diameter of adjacent normal segments, variable between studies and ranges between 1.2% and 7.4% of patients undergoing coronary angiography [1]. Histology shows advanced destruction of the musculoelastic elements with the loss of the integrity of the inner and outer elastic lamina, as well as the significant deterioration of the collagen and elastin fibers that form the main building blocks in the media layer [2]. CAE has been defined for a long time. Many factors are blamed for its etiology and pathogenesis, however, the major determinants playing a role in its etiology and pathogenesis remain unclear [2]. Among these factors, atherosclerosis is accepted as the main reason for the formation of ectasia [3]. Although it is often associated with atherosclerosis and is considered a variant of atherosclerosis, its poorer clinical outcomes than atherosclerotic CAD and the fact that it can be seen as isolated CAE without atherosclerosis suggest that determinants different from atherosclerosis may play a role in the development of CAE [4, 5]. Although patients with diabetes mellitus (DM) are a major risk factor for CAD, it is seen less frequently in these patients [6]. The precise pathophysiology of CAE still remains a mystery. Therefore, the determination of CAE determinants in DM patients in the CAD and CAE intersection cluster may shed light on the pathophysiological mechanism of this phenomenon.

C-peptide is a bioactivate molecule that is released into the blood at the same equivalent as insulin and has important effects on the vascular structure in the body [7]. There are many conflicting results in the literature regarding its negative and positive effects on vascular structure [8]. In recent years, the effect of C-peptide on the development of vascular disease and macrovascular and microvascular complications in DM has come to the fore [9]. The effect of C-peptide on type 1 and type 2 DM is clinically different. In type 1 DM characterized by insulin and C-peptide deficiency, C-peptide supplementation has been shown to improve microvascular complications [10]. In type 2 DM, C-peptide levels are generally found to be higher than normal, and high C-peptide levels have been associated with cardiovascular and all-cause mortality in patients undergoing coronary angiography [11].

These studies show that the C-peptide has a complex function. Until now, it is not known in which direction it affects CAE. In this study, we aimed to reveal the relationship between CAE and C-peptide also interaction with DM, and the effect of these factors on long-term clinical outcomes who underwent coronary angiography.

## 2. Methods

### 2.1. Study Population

Our dual-center retrospective cohort study included 6611 patients who underwent angiography between January 2013–December 2017 and underwent a mean retrospective follow-up of 60 ± 5 months. The exclusion criteria were patients with congenital heart disease, cardiomyopathy, atrial fibrillation, dilatation of less than 1.5 times the adjacent vessel, coronary artery fistula, history of autoimmune and vasculitis disease, stent-related coronary artery aneurysms, significant valvular heart disease or mechanical valve prosthesis, previous history of coronary artery bypass grafting (CABG), and patients whose national patient data are not available or missing. Angiography was performed on all patients with the suspicion of coronary artery disease using the standard Judkins technique. Patients requiring coronary revascularization were diagnosed and treated according to current guidelines. The baseline demographic, clinical data, and cardiovascular risk factors of the patients were obtained retrospectively by scanning hospital patients' medical record data. The patients were divided into two (without CAE and with CAE) according to the presence of CAE and quartiles (Q) (Q1 (C-peptide ≤ 1.46 ng/dl), Q2 (1.4 ng/dl < C-peptide ≤ 2.22), Q3 (2.22 nd/dl < C-peptide ≤ 4.06 ng/dl), Q4 (C-peptide > 4.06 ng/dl)) according to their C-peptide values. In addition, patients with CAE were divided into two groups according to the presence of DM or not, and a subgroup analysis was performed.

This retrospective study was approved by the Ethics Committee of our hospital.

### 2.2. Angiographic Assessment and Analysis

**Figure 1:**
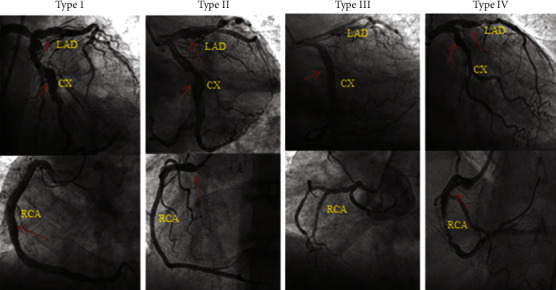
Angiographic features of ectatic coronary arteries according to the Markis classification. Type I: diffuse CAE in two or three coronary arteries (diffuse ectasia in LAD and CX and local ectasia proximal LAD). Type II: diffuse CAE in one coronary artery (CX) and localized CAE in other arteries (proximal LAD, CX). Type III: diffuse CAE in only one coronary artery (RCA). Type IV: localized or segmental CAE (proximal LAD, CX, and RCA) (red arrows point to ectatic segments). CAE, coronary artery ectasia; LAD, left anterior descending; CX, circumflex artery; RCA, right coronary artery.

Coronary angiography images of the patients were evaluated with quantitative and visual methods by two experienced interventional cardiologists. In the intervening events, a third experienced cardiologist was consulted. All dilated segments were measured and recorded in terms of maximum diameter and total length on angiographic images in Digital Imaging and Communications in Medicine (DICOM) format. CAE is defined as dilatation of the coronary lumen that exceeds the diameter of normal adjacent segments or the diameter of the patient's largest coronary artery by 1.5 times [12]. If the ectatic region involved ≥1/3 of the total vessel length, it was considered diffuse CAE, and focal CAE when it was present <1/3 [12]. According to the Markis classification (based on the topographic size of the main epicardial coronary arteries), ectatic arteries were subdivided into 4 types: type I, diffuse ectasia of at least two or three arteries; type II, diffuse ectasia in one artery and localized ectasia in the other; type III, diffuse ectasia of only one artery; type IV, localized or segmental ectatic lesions in arteries (Figure 1) [13]. Stenosis of ≥50% of ≥1 epicardial coronary artery or the main branch of the coronary arteries with a diameter >2 mm was defined as CAD, and the severity of CAD was assessed by the number of stenoses of ≥50% of the epicardial coronary arteries [14]. SYNTAX score was calculated as follows; The main epicardial coronary arteries were divided into sixteen segments according to the American Heart Association (AHA) classification. Scores assigned to each lesion identified in the epicardial coronary artery with >50% diameter stenosing in vessels >1.5 mm in diameter were added. Each segment was given 1 or 2 points according to the presence of stenotic atherosclerosis. This score was then weighted according to values ranging from 3.5 for the proximal left anterior descending artery (LAD) to 5.0 for the left main coronary artery (LMCA) and 0.5 for the smaller branches [15]. Periprocedural thrombolysis in myocardial infarction (TIMI) flow grades were recorded. Coronary artery flow was determined by using the TIMI frame count method [16]. Thrombus burden was graded from 0 to 5 according to the TIMI-thrombus scale [17]. Low thrombus burden was defined as TIMI-thrombus scale ≤3 and high thrombus burden was defined as a TIMI-thrombus scale >3.

### 2.3. Biochemical Measurement and Definitions

The biochemical parameters of the patients were obtained from the periprocedural fasting blood sample records. As the standard of our center, fasting glucose and C-peptide levels were measured in each patient who underwent angiography. Hypertension was defined as a systolic blood pressure of ≥ 140 mmHg, a diastolic blood pressure of ≥90 mmHg, or current treatment by any antihypertensive drug. The diagnosis of diabetes mellitus (DM) was based on the previous history of DM or the following criteria: fasting plasma glucose (FPG) > 126 mg/dL; HbA1c > 6.5% (48 mmol/mol); and random plasma glucose > 200 mg/dL [18]. Dyslipidemia was defined as the presence of either of the four parameters (1) total cholesterol > 200 mg/dl, (2) low-density lipoprotein (LDL) cholesterol > 130 mg/dl, (3) high-density lipoprotein (HDL) cholesterol <40 mg/dl for males and < 50 mg/dl for females, and (4) triglyceride > 150 mg/dl or previous history of statin use. Positive family history of CAD was defined as confirmed evidence of premature CAD in a first-degree relative (men < 55 and women <65 years of age). Glomerular filtration rate (GFR) calculated from Cockgroft-Gault formula ([140 age] × [weight in kg] × [0.85 if female]/[72 × creatinine]). Diagnosis, treatment, and follow-up of STEMI (ST-elevation myocardial infarction), NSTEMI (NonST-elevation myocardial infarction), unstable angina pectoris (UAP), stable angina pectoris (SAP) and Myocardial infarction with nonobstructive coronary arteries (MINOCA) were performed according to current guidelines. Myocardial infarction (MI) was defined as the detection of a rise and/or fall of troponin with at least one value above the 99^th^ percentile of the upper reference limit together with evidence of myocardial ischemia at least one of the following: symptoms of ischemia; ECG changes indicative of new ischemia (new ST-T changes or new left bundle branch block [LBBB]); Development of pathological *Q* waves in the ECG [18]. Cardiac causes were accepted unless a specific noncardiac cause of death was specified. Stroke was defined as clinical evidence of cerebral, spinal cord, or retinal focal ischemic injury based on symptoms persisting ≥ 24 hours or until death, and other etiologies excluded [19].

### 2.4. Retrospective Follow-Up and Primary End Point

Patients were followed up for an average of 56 ± 14 months. Medical and clinical retrospective follow-up data were obtained from the national health database and hospital medical patient records. Patients whose medical records could not be reached were excluded from the study (In total, 128 of 6739 patients included in the study were excluded from the study due to lack of medical data records. The study was completed with a total of 6611 patients.). The primary endpoint was 60-month composite major adverse cardiovascular events (MACE), which was a component of myocardial infarction (MI), cardiac death, and stroke. Individual components of MACE were accepted as a secondary outcome.

### 2.5. Statistical Analysis

Statistical Program for Social Sciences 20 (IBM SPSS, Chicago, IL, USA) was used for all statistical calculations. Kolmogorov-Smirnov test was used to determine whether the data fit the normal distribution. Continuous variables that fit the normal distribution were expressed as means ± standard deviation (SD), and those that did not fit the normal distribution were expressed as median with interquartile range (IQR). Comparisons between CAE, DM groups, and other subgroups were analyzed using the Mann–Whitney *U* test, independent *t*-test, and one-way analysis of variance (ANOVA), where appropriate. The Chi-square test was applied to categorical variables. Pearson correlation coefficient was used to determine the relationship between C- peptide levels and maximum ectatic diameter and total length of an ectatic segment. Univariate and multivariate regression analyses were performed to determine the independent predictors of CAE. Baseline variables with significant significance (*p* < 0.05) by univariate analysis were included in the multivariate logistic regression analysis. Receiver operating characteristic (ROC) curve analysis was performed to determine and compare of C-Peptide and other parameters found to be independent predictors of CAE in previous studies. The optimal cut-off value was calculated from the point of maximum sensitivity and specificity (Youden's index). The cumulative events were evaluated using the Kaplan–Meier curves and compared using the log-rank test. Univariate and multivariate Cox proportional hazards model analysis was used to evaluate the association between CAE, C-peptide, DM, and MACE. Results of Cox regression analysis were presented as hazard ratio (HR) and 95% confidence interval (CI). Two-tailed *p*-values of <0.05 were considered to be statistically significant.

## 3. Results

**Figure 2:**
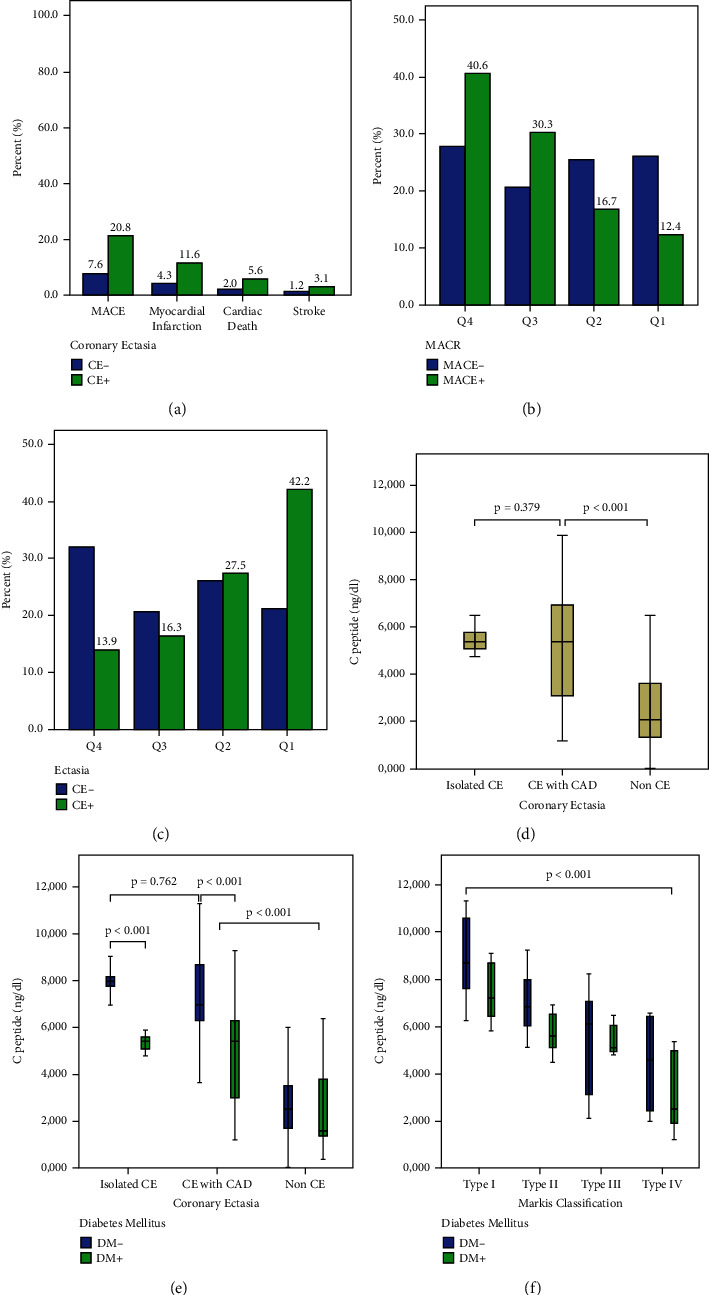
Distribution of individual MACE in patients with and without CAE during follow-up. (b) MACE percentages by C-peptide quartiles. (c) Percentages of coronary ectasia by C-peptide quartiles. (d) Mean C-peptide levels of isolated CAE, CAE with CAD, and non-CAE patient groups. (e) C-peptide levels in isolated CAE, CAE with CAD, and non-CAE patient groups according to DM or not. (f) C-peptide levels according to the presence or absence of DM in the patient groups separated according to the Markis classification. CA, coronary artery ectasia; MACE, major adverse cardiovascular event.

**Figure 3:**
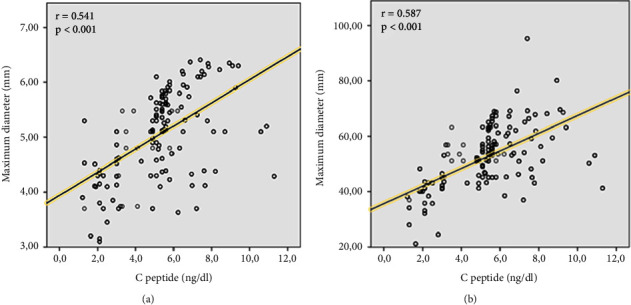
Correlation analysis between C-peptide levels and maximum ectatic segment diameter (a) and maximum ectatic segment length (b).

**Figure 4:**
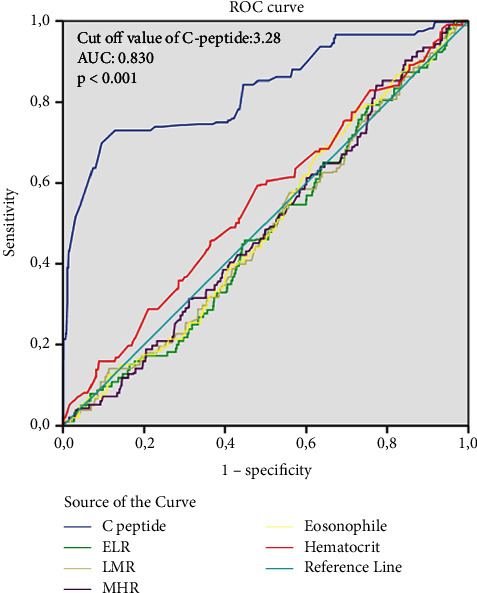
ROC curve analysis. ROC curve analysis showed that at a cut-off of 3.28 ng/dl, the value of C-peptide exhibited 76% sensitivity and 60% specificity for predicting coronary artery ectasia (area under the curve [AUC] = 0.830, *p* < 0.001).

In total, CAE was detected in 552 (8.3%) of 6611 patients. The mean age was 58.83 ± 12.89 years and 368 (66.4%) of them were male. The baseline demographic, clinical, and laboratory parameters of the patients are given in Table 1. They presented a higher prevalence of hypertension, dyslipidemia, smoking status, and STEMI diagnosis, but a lower prevalence of DM. In addition, triglyceride, total cholesterol (TC), C-reactive protein (CRP), white blood cell count (WBC), basophil count, hematocrit (Hct), C-peptide level, and severity of CAD were higher in the group with CAE; fasting plasma glucose (FPG), uric acid, albumin, and high-density lipoprotein cholesterol (HDL-C) amount were higher (*p* < 0.05, for all). There was no significant difference in other baseline demographic, clinical, and laboratory parameters (*p* > 0.05). As seen in Table 2 in the angiographic analysis of the patients culprit lesion was seen mostly in the right coronary artery (RCA) (51.1%) and least in the left main coronary artery (LMCA) (1.3%). The three-vessel disease was more common in the non-CAE group and thrombus burden, maximum stent diameter, preprocedural, and postprocedural low thrombolysis in myocardial infarction (TIMI) flow grade (0/1) were higher in CAE group (*p* < 0.05, for all). Coronary ectasia was isolated in 13.8% of patients and it was associated with CAD in 86.2% (Table 3). Also, CAE was seen at 52.7% in RCA, 39.6% in the left anterior descending artery (LAD), 5.5% in the circumflex artery (CX), and 2.2% in LMCA (Table 4). Of the patients with CAE, 9% were in the Markis type I, 20% in the Markis type II, 35.8% in the Markis type III, and 34.6% in the Markis type IV class (Table 4). Patients with CAE were further divided into subgroups according to the presence of DM. Baseline demographic, clinical, and laboratory parameters of coronary ectasia patients with and without DM are presented in Table 3 and their angiographic features are presented in Table 4. Female gender and hypertension were more common in the diabetic group. Also, in the diabetic group higher in FPG, creatinine, uric acid, Triglyceride, TC, low-density lipoprotein cholesterol (LDL-C), mean platelet volume (MPV), gamma-glutamyl transferase (GGT), HbA1c, C-peptide and albumin, HDL-C, and total bilirubin were significantly lower (*p* < 0.05, for all) (Table 3). MACE developed in 20.8% of patients with CAE and 7.2% of patients without CAE during a 60-month follow-up (*p* < 0.001). Myocardial infarction, cardiac death, and stroke rates were significantly higher than in patients without CAE (11.6% vs 4.3%, 5.6% vs 2.0%, 3.1% vs 1.2%; *p* < 0.001, respectively) (Figure 2(a)). As C-peptide levels (quartiles) increased, MACE and ectasia rates increased (Q4: 40.6%, Q3: 30.3%, Q2: 16.7%, Q1: 12.4%; Q4: 42.2%, Q3: 27.5%; Q2: 16.3%, Q1: 13.9%; *p* < 0.001, respectively) (Figures 2(b) and 2(c)). Although C-peptide levels were significantly higher in patients with isolated CAE and CAE with CAD than in patients without ectasia (*p* < 0.001), there was no statistically significant difference between isolated CAE and CAE with CAD (*p*=0.379) (Figure 2(d)). C-peptide levels were significantly lower in diabetic patients with both isolated CAE and CAE with CAD than in nondiabetic patients (*p* < 0.001); however, they were significantly higher than in diabetic patients without CAE (*p* < 0.001). In addition, there was no significant difference in C-peptide levels in ectasia patients with isolated CAE and CAE with CAD and in patients with ectasia with isolated nondiabetic CAE and CAE with CAD (*p*=0.128, *p*=0.762, respectively) (Figure 2(e)). C-peptide levels were higher in type I and type II, where vascular involvement was more common, compared to type III and type IV, according to the Markis classification (*p* < 0.001) (Figure 2(f)). There was a positive correlation between C-peptide levels and maximum ectatic segment diameter and maximum ectatic segment length (*r* = 0.541, *p* < 0.001; *r* = 0.587, *p* < 0.001, respectively) (Figure 3). During a mean follow-up of 56 ± 14 months, 573 patients (8.7%) had MACE. Of these, 322 (4.9%) had a myocardial infarction, 153 (2.3%) had cardiac death, and 92 (1.4%) had a stroke. In multivariate regression analysis, C-peptide and Hct elevation were determined as independent predictors for the presence of CAE (odds ratio: 2.417, 95% CI: 2.212–2.641, *p* < 0.001; odds ratio: 1.035, 95% CI: 1.022–1.048, *p* < 0.001, respectively) (Table 5). After adjusting for parameters that turned out to be significant in the univariate analysis, DM associated with a 0.27-fold (95% CI: 0.191–0.382, *p* < 0.001) reduced the likelihood of CAE on coronary angiography. Receiver operating characteristic analysis showed that C-peptide values of ≥ 3.28 could predict CAE with a sensitivity of 76% and specificity of 60% for predicting CIN (area under the curve (AUC) = 0.830, *p* < 0.001) (Figure 4). In addition, while this analysis was significant for Hct (AUC: 0.540, *p* < 0.001), it was found to be associated with CAE in the literature; inflammation markers such as eosinophil/lymphocyte ratio, lymphocyte/monocyte ratio, and monocyte/HDL ratio were not found to be significant (*p* > 0.05, for all) (Figure 4).

In the multivariate Cox regression analysis, the presence of CAE, high C-peptide, and HbA1c, low albumin levels were determined as independent predictors for MACE development (hazard ratio: 1.012, 95% CI: 1.002–1.023, *p*=0.026; hazard ratio: 1.255, 95% CI: 1.164–1.336, *p* < 0.001; hazard ratio: 1.225, 95% CI: 1.028–1.461, *p*=0.024; hazard ratio: 0.632, 95% CI: 0.409–0.975, *p*=0.038, respectively) (Table 6). In the patient group with CAE, DM was not associated with the development of MACE in the cox regression analysis, in addition, C-peptide was found to be an independent predictor for the development of MACE in the subgroup of patients with CAE (Hazard Ratio: 1.254, 95% CI: 1.212–1.354, *p* < 0.001) (Table 7).

**Figure 5:**
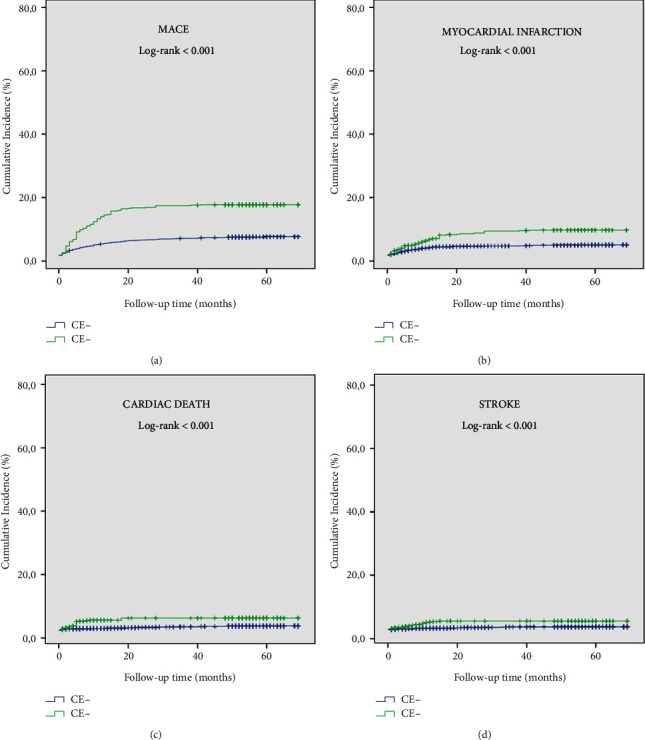
Kaplan–Meier survival curves of cumulative incidence of (a) MACE, (b) myocardial infarction, (c) cardiac death, and (d) stroke in patients with CAE (green line) versus patients without CAE (blue line); MACE, major adverse cardiovascular event; CAE, coronary artery ectasia.

**Figure 6:**
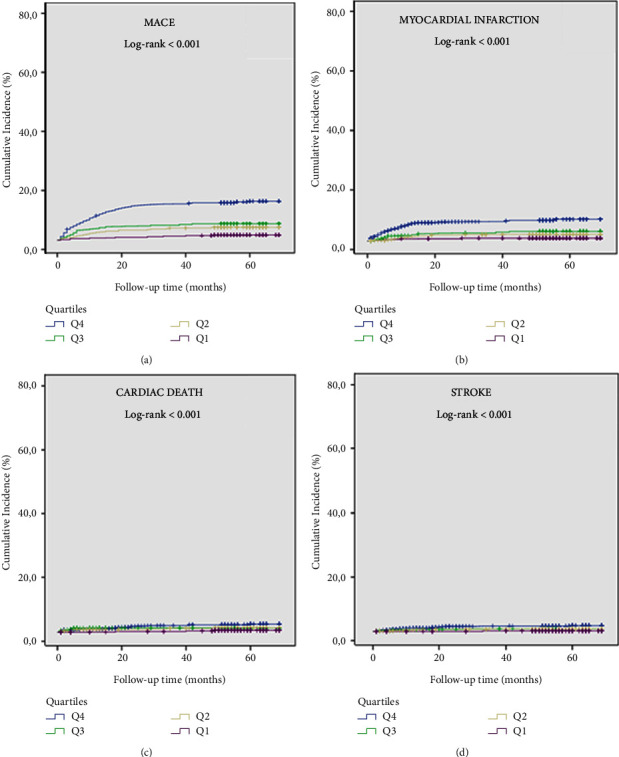
Kaplan–Meier survival curves of cumulative incidence of (a) MACE, (b) myocardial infarction, (c) cardiac death, and (d) stroke in patients according to their C-peptide quartiles (Q4-blue line; Q3-green line; Q2-yellow line; Q1-Purple line); MACE, major adverse cardiovascular event.

**Figure 7:**
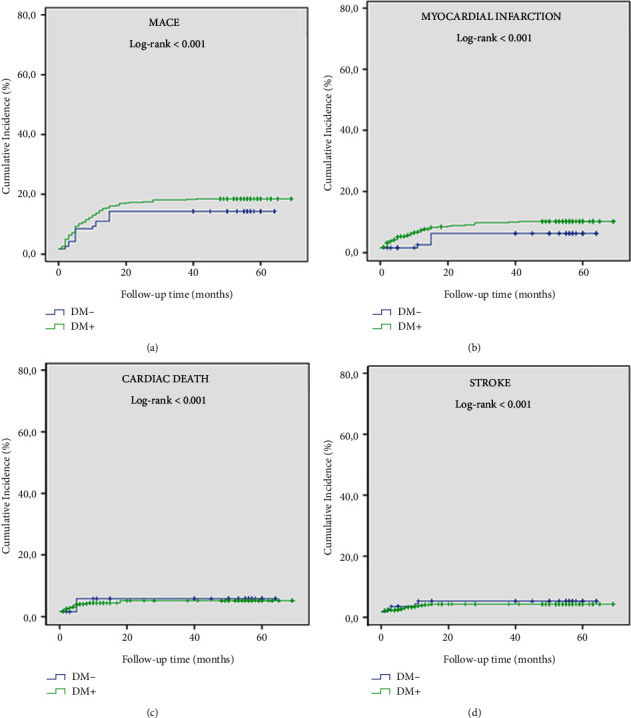
Kaplan–Meier curves showed similar risks of MACE (a), myocardial infarction (b), cardiac death (c), and stroke (d) among the DM group and non-DM group.

**Figure 8:**
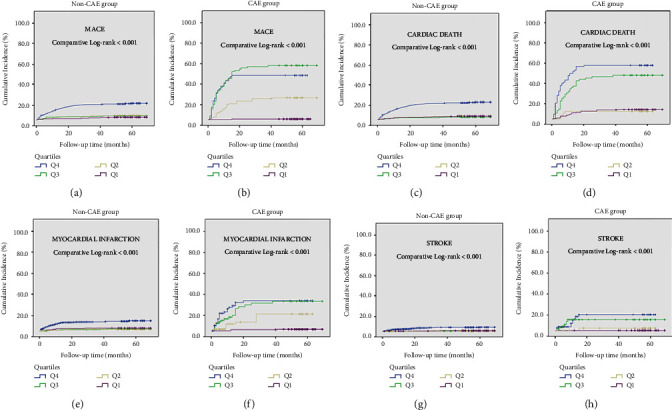
Kaplan–Meier survival curves of cumulative incidence of MACE (a-b); cardiac death (c-d) myocardial infarction (e-f); and stroke (g-h) in patients with CAE and without CAE according to their C-peptide quartiles (Q4-blue line; Q3-green line; Q2-yellow line; Q1-purple line); CAE: coronary artery ectasia; MACE: major adverse cardiovascular event.

In Kaplan–Meier survival curve analysis, the cumulative incidence of MACE and its individual components in patients with CAE was significantly higher than in patients without CAE (log-rank < 0.001, for all) (Figure 5). In addition, there was no significant difference between diabetic and nondiabetic patients in terms of cumulative incidence of MACE and its individual components in patients with CAE (log-rank > 0.05, for all) (Figure 6). In the Kaplan–Meier survival curve analysis, the cumulative incidence of MACE and its individual components at Q4 was significantly higher than in the other quartiles (log-rank < 0.001, for all) (Figure 7). Furthermore, the cumulative incidence of MACE and its components was significantly higher in the Q3 and Q4 quartiles than in the Q1 and Q2 quartiles in both CAE and non-CAE patients, although this difference was significantly greater in patients with CAE than in patients without CAE (log-rank < 0.001) (Figure 8).

## 4. Dicussion

In this study, we basically wanted to reveal the effect of C-peptide on CAE and the interaction of these two parameters in diabetic patients, as well as the composite effect of C-peptide, CAE, and diabetes triad on long-term clinical outcomes in patients who underwent coronary angiography. In our study, we revealed that C-peptide is an independent predictor of CAE and that these two parameters may be significantly related to long-term clinical outcomes. To the best of our knowledge, this is the first study to examine the relationship between C-peptide and CAE and to investigate the composite effect of these two parameters on long-term clinical outcomes.

Although CAE is as common as stenotic coronary artery diseases in routine practice, it is of important clinical importance because of its close relationship with cardiovascular diseases. Many mechanisms are implicated in the underlying etiology of CAE however, what triggers this process has not yet been fully clarified [2]. It is frequently associated with atherosclerosis and the underlying risk factors similar to atherosclerosis have led to the evaluation of this phenomenon as a variant of atherosclerosis [20]. In addition to atherosclerosis, a close relationship has been found with some vasculitis and autoimmune diseases [21]. It has also been found to be associated with causes such as familial hypercholesterolemia, drug-eluting stent, abdominal aortic aneurysm, and inflammation [22–25]. Although CAE is considered to be an atherosclerotic process, it is interestingly less common in diabetes patients, which is considered a major atherosclerosis risk factor [26]. The fact that two major atherosclerotic diseases are at different extremes in this respect made us think that there are processes that interact in common between the two. C-peptide is a molecule that diffuses into the blood on an equal basis with insulin and is often used in current practice to estimate the insulin reserves of diabetic patients. Studies have shown that C-peptide has a highly active and complex role in human physiology, but its mechanism of action is not yet fully understood. The effects of C-peptide vary depending on the target tissue, pathophysiological conditions, interaction with bioactive molecules, or concentration [6]. We detected C-peptide as an independent risk factor for CAE in our study. Although the mechanism of action is not clear, this interaction may be due to the proatherogenic effects of the C-peptide because atherosclerosis appears to be a major risk factor for the development of CAE [12]. A study by Marx et al. found that C-peptide stimulates the migration of inflammatory cells to the lesion in the early stages of the atherosclerotic lesion, which in turn induces smooth muscle cell proliferation [27]. Other experimental studies have shown that C-peptide induces the progression of atherosclerosis, while *in vitro* studies induce migration of vascular smooth muscle cells and infiltration of inflammatory cells [28, 29]. Matrix metalloproteinases play an important role in the development of CAE [30]. Wang et al. found that C-peptide induces matrix metalloproteinase 9 (MMP9) in mesangial cells in an experimental mouse model [31]. C-peptide-mediated stimulation of mitogen-activated protein kinases (MAPKs) has been demonstrated in previous studies and MAPK signal-mediated induction of MMP2, which plays a role in the pathogenesis of CAE, may contribute to the development of CAE [30, 32, 33]. In another study, it was found that C-peptide inhibited the synthesis of collagen type IV (COL4), which is one of the building blocks of the extracellular matrix [34]. The angiogenesis process has been also implicated in the pathogenesis of CAE [35, 36]. In a study by Lim et al., C-peptide was found to accelerate angiogenesis through stimulation of extracellular signal-regulated kinase (ERK 1/2), Akt, and NO production using human umbilical vein endothelial cells (HUVEC) [37]. Coronary artery calcification is increased in patients with coronary ectasia [38, 39]. This suggests that coronary artery calcification may play a role in the pathogenesis of CAE, and in a study by Burgmaier et al., C-peptide was found to be an independent predictor of coronary artery calcification [40]. Nitric Oxide (NO) plays an important role in the pathogenesis of CAE. It has been determined that endothelium-mediated stimulation of inducible NO synthase (i-NOS) synthesis stimulates the expression of MMPs, which play an important role in the development of CAE [41]. In addition, NO degradation products such as peroxynitrate and nitrate have been found to induce MMP activation, which may contribute to the pathogenesis of CAE [42]. In addition, in an experimental study, it was shown that NO, specially synthesized from the i-NOS pathway, plays a major role in the development of CAE [43]. In addition, it has been shown in previous studies that the inflammatory process and adhesion molecules play a critical role in the pathogenesis of CAE [44, 45]. In a study by Janowska et al., it was determined that C-peptide correlated positively with E-selectin and negatively correlated with IL-10, which has anti-inflammatory properties [46]. These results indicate the possible active involvement of higher C-peptide concentrations in the development of ectasia in patients with CAE. In a meta-analysis, low C-peptide levels were associated with cardiovascular mortality and the development of cerebrovascular events in diabetic patients48, while in other studies, high C-peptide levels were associated with poor long-term clinical outcomes [10, 47, 48]. In addition, CAE was associated with increased cardiovascular mortality [3, 5]. In our study, C-peptide is independently associated with precense of CAE and C-peptide levels were positively correlated with the severity of CAE also there was a negative association between diabetes mellitus and the presence of CAE. CAE and C-peptide were found to be independent predictors of increased cardiovascular mortality, in addition, the development of cardiovascular events in patients with high C-peptide and CAE suggested that the composite effect of these two factors may have synergistic effects on clinical outcomes. Based on these findings, further studies incorporating our current data may shed light on the pathogenesis of CAE and perhaps assist in the development of new pharmacological models to reduce the chances of vessel wall injury and poor clinical outcomes.

### 4.1. Limitations

This study had several limitations. First, this was an observational retrospective analysis of prospectively clinically obtained data. Second, percutaneous coronary intervention optimization with intracoronary imaging was not routinely applied, which may have affected clinical outcomes. Third, the ectatic patient group had a relatively low sample size. Finally, diabetic patients were not separated according to their types, which may cause differences in clinical outcomes.

## 5. Conclusions

In this study, we found that C-peptide is independently associated with precense of CAE and there is a positive correlation between C-peptide and CAE severity and there is also a significant negative association between diabetes and CAE. In addition, we showed that C-peptide and CAE composite can have a synergistic effect on poor clinical outcomes. Larger prospective studies are needed to confirm the results of this study [49].

## Figures and Tables

**Table 1:** Distribution of clinical and demographic characteristics of the patients according to coronary artery ectasia.

Variables	Total population (*n* = 6611)	Without CAE (*n* = 6059)	With CAE (*n* = 552)	*p* value
Demographics and medical history				
Age (years)	60.27 ± 12.23	60.42 ± 12.15	58.83 ± 12.89	0.002
Gender, male, *n* (%)	4515 (68.2)	4147 (68.4)	368 (66.6)	0.565
BMI (kg/m^2^)	26.4 ± 2.83	24.08 ± 3.20	25.45 ± 3.39	0.310
Diabetes mellitus, *n* (%)	2259 (34.1)	2167 (55.6)	92 (20.0)	<0.001
Hypertension, *n* (%)	4415 (66.7)	4114 (67.8)	301 (54.5)	<0.001
Dyslipidemia, *n* (%)	5370 (81.2)	5017 (82.8)	353 (63.9)	<0.001
Smoking, *n* (%)	4059 (61.3)	3743 (61.7)	316 (57.2)	0.036
LVEF (%)	50.3 (51.0–59.0)	51.0 (45.3–54.0)	50.2 (41.0–53.0)	0.115
Family history of CAD	793 (11.9)	725 (11.9)	68 (12.3)	0.583
Prior CAD	613 (9.2)	552 (9.1)	59 (10.6)	0.136

Diagnosis, *n* (%)				
MINOCA	243 (3.7)	188 (3.1)	55 (10)	<0.001
NSTEMI	3026 (45.8)	2596 (48.8)	70 (12.7)	
AWSTEMI	1000 (15.1)	852 (14.1)	148 (26.8)	
PWSTEMI	947 (14.3)	787 (13.0)	160 (29.0)	
UAP	414 (6.3)	381 (6.3)	33 (6.0)	
SAP	981 (14.8)	895 (14.8)	86 (15.6)	

Laboratory results				
FPG (mg/dL)	122 (98.0–172.0)	120 (98.0–170.0)	110.0 (93.0–142.0)	<0.001
Creatinine (mg/dL)	0.80 (0.70–1.00)	0.80 (0.70–1.00)	0.80 (0.70–1.00)	0.757
Uric acid (mg/dL)	5.10 (4.30–6.10)	5.20 (4.20–6.10)	5.0 (4.10–6.00)	<0.001
Albumin (mg/dL)	4.20 (3.90–4.40)	4.30 (4.00–4.50)	4.10 (3.90–4.40)	<0.001
Triglycerides (mg/dL)	146.0 (101.0–214.0)	142.0 (101.0–201.0.)	151 (106.0–278.0)	0.035
TC (mg/dL)	179.0 (147.0–211.0)	178 (144.0–205.0)	182.0 (153.0–226.0)	0.011
HDL-C (mg/dL)	35.0 (28.0–42.0)	35.0 (30.0–41.0)	33 (27.0–38.0)	<0.001
LDL-C (mg/dL)	109.45 ± 37.41	109.71 ± 37.09	106.96 ± 40.26	0.080
CRP (mg/dL)	0.51 (0.18–1.21)	0.52 (0.20–1.18)	0.58 (0.21–1.26)	<0.001
eGFR (ml/min)	87.15 ± 20.73	87.00 ± 20.59	88.55 ± 21.99	0.075
WBC (×1000/mm^3^)	9.60 (7.66–12.40)	9.48 (7.60–12.10)	10.03 (7.65–12.75)	0.010
Lymphocyte (×1000/mm^3^)	2.00 (1.40–2.60)	2.00 (1.47–2.60)	2.00 (1.43–2.70)	0.956
Monocytes (×1000/mm^3^)	0.60 (0.47–0.72)	0.60 (0.46–0.81)	0.60 (0.44–0.80)	0.303
Neutrophil (×1000/mm^3^)	6.31 (4.55–8.06)	7.24 (4.50–8.80)	6.70 (4.60–9.85)	0.118
Eosinophil (×10^9^ cells/l)	0.093 (0.034–0.190)	0.093 (0.033–0.019)	0.092 (0.049–0.183)	0.251
Basophil (×10^9^ cells/l)	0.027 (0.008–0.061)	0.026 (0.008–0.059)	0.030 (0.011–0.080)	<0.001
RDW (fL)	12.7 (12.00–13.50)	12.7 (12.0–13.5)	12.9 (12.2–13.7)	0.390
MPV (fL)	7.97 (7.21–8.76)	7.97 (7.20–8.80)	8.08 (7.40–8.85)	0.130
Calcium (mg/dL)	9.10 (8.70–9.50)	9.10 (8.70–9.50)	9.10 (8.80–9.50)	0.354
ALT (U/L)	24.0 (18.0–35.0)	24.0 (18.0–36.0)	23.0 (17.0–34.0)	0.100
AST (U/L)	30 (22.0–47.0)	30.0 (24.0–50.0)	29.1 (20.0–40.0)	0.215
ALP (U/L)	85.28 ± 26.40	85.61 ± 26.66	82.46 ± 23.97	0.009
GGT (U/L)	26.0 (18.0–45.0)	26.0 (18.0–44.0)	29.0 (18.0–45.0)	0.631
Hemoglobin (mg/dL)	14.10 ± 4.38	14.13 ± 4.57	14.06 ± 1.89	0.482
Hematocrit, (%)	43.0 (39.30–47.0)	43.0 (39.0–47.0)	44.0 (40.0–48.0)	0.001
Platelet count (×1000/mm^3^)	259.0 (217.0–309.0)	259.0 (215.0–309.0)	257.0 (223.0–308.0)	0.725
Hemoglobin A1c (mg/dL)	6.05 (5.50–7.30)	5.90 (5.10–6.50)	6.00 (5.40–6.20)	0.086
C-peptide (ng/dL)	2.78 ± 1.72	2.50 ± 1.39	5.42 ± 2.25	<0.001
Eosonophil/lymphocyte ratio	0.044 (0.019–0.084)	0.044 (0.018–0.085)	0.043 (0.021–0.076)	0.717
Lymphocyte/monocyte ratio	3.28 (2.30–4.52)	3.28 (2.28–4.55)	3.25 (2.38–4.33)	0.842
Monocyte/HDL ratio	0.018 (0.012–0.026)	0.018 (0.012–0.026)	0.017 (0.012–0.025)	0.139
Severity of CAD	1.77 ± 1.01	1.79 ± 1.00	1.57 ± 1.03	<0.001

Medications at discharge				
Antiplatelet	6510 (98.4)	5985 (98.7)	548 (95.1)	0.124
*β*-blocker	5810 (87.8)	5321 (87.8)	489 (88.7)	0.325
Statins	6413 (97.0)	5877 (96.9)	536 (97.1)	0.348
CCB	2783 (42.0)	2549 (42.0)	234 (42.3)	0.453
ACEI/ARB	3966 (59.9)	3645 (60.1)	321 (58.1)	0.225
Nitrates	991 (14.9)	920 (15.1)	71 (12.8)	0.080
ARA	462 (6.9)	421 (6.9)	41 (7.4)	0.114

Values are mean ± SD, *n* (%), or median (interquartile range) unless otherwise stated. ACEI: angiotensin-converting enzyme inhibitors; ALT: alanine aminotransferase; AST: aspartate aminotransferase; ALP: alkaline phosphatase; ARB: angiotensin receptor blockers; ARA: aldosterone receptor antagonist; AWSTEMI: anterior wall STEMI; PWSTEMI: posterior wall STEMI; BMI: body mass index; CAD: coronary artery disease; CAE: coronary artery ectasia; CCB: calcium channel blocker; CRP: C- reactive protein; FPG: fasting plasma glucose; GGT: gamma-glutamyl transferase; LVEF: left ventricular ejection fraction; MINOCA: myocardial infarction with nonobstructive coronary arteries; UAP: unstable angina pectoris; NSTEMI: non-ST-segment elevation myocardial infarction; STEMI: ST-Segment elevation myocardial infarction; SAP: stable angina pectoris; PCI/PTCA: percutaneous coronary intervention/percutaneous transluminal coronary angioplasty; eGFR: estimated glomerular filtration rate; TC: total cholesterol; HDL-C: high-density lipoprotein cholesterol; LDL-C: low-density lipoprotein cholesterol; MPV: mean platelet volume; RDW: red cell distribution width; WBC: white blood cell.

**Table 2:** Angiographic and procedural characteristics of the study population.

Variables	Total population (*n* = 6611)	Without CAE (*n* = 6059)	With CAE (*n* = 552)	*p* value
Severity of CAD (%)				
None	878 (13.3)	760 (12.5)	118 (21.4)	<0.001
Single-vessel	1693 (25.6)	1519 (25.1)	174 (31.5)	
Double-vessel	2104 (31.8)	1910 (31.5)	194 (35.1)	
Three-vessel	1936 (29.3)	1870 (30.9)	66 (12.0)	
SYNTAX score	13.0 (8.0–20.0)	14.0 (8.0–19.0)	13.0 (7.0–21.0)	0.089

Culprit lesion location (%)				
LAD artery	2488 (37.6)	2315 (38.2)	166 (30.1)	<0.001
CX artery	1248 (18.9)	1151 (19.0)	97 (17.6)	
RCA	2798 (42.3)	2516 (41.5)	282 (51.1)	
LMCA	77 (1.3)	77 (1.3)	7 (1.3)	
PCI/PTCA	5409 (81.8)	4961 (81.8)	448 (81.1)	0.545

Thrombus burden				
Low thrombus burden	2724 (41.2)	2582 (42.6)	142 (25.7)	<0.001
High thrombus burden	3887 (58.8)	3477 (57.4)	462 (74.3)	
Thrombectomy	553 (8.3)	506 (8.3)	47 (8.4)	0.318
Max. stent diameter (mm)	3.3 (3.0–3.7)	3.0 (2.75–3.50)	3.5 (3.0–4.0)	<0.001
Total stent length (mm)	24 (20–33)	24 (20–36)	23 (22–33)	0.142

Preprocedural TIMI flow				
0/1	5231 (79.1)	4713 (77.8)	491 (88.9)	<0.001
2	1031 (15.6)	1005 (16.6)	26 (4.7)	
3	349 (5.3)	341 (5.6)	35 (6.3)	

Postprocedural TIMI flow				
0/1	87 (1.3)	82 (1.4)	5 (0.9)	<0.001
2	464 (7.0)	366 (6.0)	98 (17.8)	
3	6060 (91.7)	5611 (92.6)	449 (81.3)	

CAD: coronary artery disease; CAE: coronary artery ectasia; LAD: left anterior descending; LMCA: left main coronary artery; CX: circumflex; RCA: right coronary artery; PCI/PTCA: percutaneous coronary intervention/percutaneous transluminal coronary angioplasty; TIMI: thrombolysis in myocardial infarction.

**Table 3:** Baseline characteristics of coronary ectasia patients according to their diabetic status.

Variables	Total population *n* = 552	Non-DM group *n* = 460	DM group *n* = 92	*p* value
Demographics and medical history				
Age (years)	57.55 ± 12.80	57.02 ± 12.68	60.45 ± 13.44	0.120
Gender, male, *n*(%)	368 (66.7)	317 (68.8)	51 (56.0)	0.007
BMI (kg/m^2^)	23 (18–25)	22 (18–24)	24 (19–26)	0.128
Hypertension, *n*(%)	284 (51.4)	227 (49.2)	57 (62.6)	0.022
Dyslipidemia, *n*(%)	332 (60.1)	279 (60.5)	53 (58.2)	0.685
Smoking, *n*(%)	316 (57.2)	269 (58.4)	47 (51.6)	0.158
LVEF, (%)	51 (45–58)	48 (46–60)	53 (44–55)	0.340

Diagnosis, *n*(%)				
MINOCA	46 (8.3)	40 (8.7)	6 (6.6)	0.085
NSTEMI	70 (12.7)	56 (12.1)	14 (15.4)	
AWSTEMI	148 (26.8)	119 (25.8)	29 (31.9)	
PWSTEMI	169 (30.6)	138 (29.9)	31 (34.1)	
UAP	33 (6.0)	27 (5.9)	6 (6.6)	
SAP	86 (15.6)	81 (17.6)	5 (5.5)	

Coronary artery ectasia distributions, *n* (%)				
Isolated CAE, *n*(%)	76 (13.8)	69 (15.0)	7 (7.7)	0.068
CAE with CAD, *n*(%)	476 (86.2)	392 (85.0)	84 (92.3)	

Laboratory results				
FPG (mg/dL)	127.1 ± 63.2	107.6 ± 23.6	224.5 ± 99.5	<0.001
Creatinine (mg/dL)	0.92 ± 0.50	0.90 ± 0.52	1.06 ± 0.32	0.027
Uric acid (mg/dL)	5.43 ± 1.46	5.39 ± 1.49	5.60 ± 1.27	0.035
Albumin (mg/dL)	4.31 ± 0.48	4.32 ± 0.49	4.26 ± 4.27	0.664
Triglycerides (mg/dL)	144.0 (99.0–193.0)	135.0 (95.0–181.0)	141.0 (135.0–198.0)	<0.001
TC (mg/dL)	174.1 ± 46.1	171.0 ± 43.4	196.9 ± 58.1	<0.001
HDL-C (mg/dL)	38.4 ± 11.9	38.9 ± 12.2	35.7 ± 9.5	0.297
LDL-C (mg/dL)	106.4 ± 39.5	104.5 ± 37.4	115.7 ± 48.1	0.004
CRP (mg/dL)	0.42 (0.16–0.94)	0.43 (0.16–0.98)	0.42 (0.15–0.75)	0.138
eGFR (mL/min)	89.9 ± 22.1	92.9 ± 20.1	74.8 ± 25.3	<0.001
WBC (×1000/mm^3^)	9.50 (7.2–11.9)	9.20 (7.25–11.5)	12.2 (7.7–16.5)	0.067
Lymphocyte (×1000/mm^3^)	2.10 (1.60–2.60)	2.10 (1.60–2.70)	2.10 (1.50–2.42)	0.089
Monocytes (×1000/mm^3^)	0.62 (0.49–0.82)	0.60 (0.47–0.80)	0.80 (0.50–0.91)	0.216
Neutrophil (×1000/mm^3^)	6.10 (4.40–8.50)	5.90 (4.30–7.70)	8.90 (5.40–14.0)	0.410
Eosinophil (×10^9^ cells/l)	0.094 (0.046–0.171)	0.091 (0.050–0.171)	0.118 (0.045–0.146)	0.436
Basophil (×10^9^ cells/l)	0.030 (0.011–0.082)	0.030 (0.010–0.084)	0.023 (0.012–0.078)	0.078
RDW (fL)	12.75 ± 1.69	12.67 ± 1.75	13.2 ± 0.97	0.268
MPV (fL)	8.09 ± 1.35	8.00 ± 1.31	8.56 ± 1.46	0.031
Total bilirubin (mg/dL)	0.70 ± 0.32	0.67 ± 0.33	0.62 ± 0.22	0.044
Phosphorus (mg/dL)	3.08 ± 0.77	3.08 ± 0.61	3.06 ± 1.34	0.251
Calcium (mg/dL)	9.09 ± 0.60	9.09 ± 0.59	9.08 ± 0.72	0.985
ALT (U/L)	23.0 (17.0–34.0)	21.0 (17.0–33.0)	31.0 (20.0–43.0)	0.371
AST (U/L)	25.0 (20.0–40.0)	25.0 (20.0–40.0)	26.0 (21.0–53.0)	0.420
ALP (U/L)	81.46 ± 2 4.1	81.28 ± 23.42	82.73 ± 28.5	0.883
GGT (U/L)	28.5 (18.0–45.0)	27.0 (17.2–45.0)	33.0 (20.0–50.7)	0.036
Hemoglobin (mg/dL)	14.12 ± 1.89	14.21 ± 1.85	13.63 ± 2.00	0.113
Hematocrit (%)	45.06 ± 13.06	45.62 ± 13.98	42.24 ± 6.17	0.078
Platelet count (×1000/mm^3^)	262.5 (218.0–312.0)	268.0 (219.0–313.0)	256.0 (195.0–306.0)	0.345
Hemoglobin A1c (mg/dL)	6.01 ± 1.63	5.52 ± 0.48	8.46 ± 2.77	<0.001
C-peptide (ng/dL)	5.30 ± 2.08	4.99 ± 1.77	6.85 ± 2.73	<0.001

Values are mean ± SD, *n* (%), or median (interquartile range) unless otherwise stated. ALT: alanine Aminotransferase; AST: aspartate aminotransferase; ALP: alkaline phosphatase; AWSTEMI: anterior wall STEMI; PWSTEMI: posterior wall STEMI; BMI: body mass index; CAD: coronary artery disease; CAE: coronary artery ectasia; CCB: calcium channel blocker; CRP: C- reactive protein; FPG: fasting plasma glucose; GGT: gamma-glutamyl transferase; LVEF: left ventricular ejection fraction; MINOCA: myocardial infarction with nonobstructive coronary arteries; UAP: unstable angina pectoris; NSTEMI: non-ST-segment elevation myocardial infarction; STEMI: ST-segment elevation myocardial infarction; SAP: stable angina pectoris; PCI/PTCA: percutaneous coronary intervention/percutaneous transluminal coronary angioplasty; eGFR: estimated glomerular filtration rate; TC: total cholesterol; HDL-C: high-density lipoprotein cholesterol; LDL-C: low-density lipoprotein cholesterol; MPV: mean platelet volume; RDW: red cell distribution width; WBC: white blood cell.

**Table 4:** Angiographic characteristics of coronary artery ectasia patients according to their diabetic status.

	Non-DM group *n* = 460	DM group *n* = 92	*p* value
Severity of CAD (%)			
None	103 (22.3)	15 (16.5)	0.294
Single-vessel	148 (32.1)	26 (28.6)	
Double-vessel	152 (33.0)	42 (46.2)	
Three-vessel	58 (12.6)	8 (8.8)	
SYNTAX score	12.00 (8.00–23.00)	14.00 (9.00–26.50)	0.035

Culprit lesion location (%)			
LAD artery	145 (31.5)	21 (23.1)	0.437
CX artery	74 (16.1)	23 (25.3)	
RCA	236 (51.2)	46 (50.5)	
LMCA	6 (1.3)	1 (1.1)	
PCI/PTCA	370 (80.3)	70 (23.1)	0.469

CAE affected coronary artery, *n* (%)			
LAD artery	146 (31.7)	36 (39.6)	
CX artery	79 (17.1)	5 (5.5)	
RCA	222 (48.2)	48 (52.7)	
LMCA	14 (3.0)	2 (2.2)	
CAE in the culprit artery	372 (80.7)	72 (79.1)	0.730

Thrombus burden			
Low thrombus burden	121 (26.2)	21 (23.1)	0.527
High thrombus burden	340 (73.8)	70 (76.9)	
Thrombectomy	33 (7.1)	6 (6.5)	0.125
Max. ectasia diameter (mm)	5.1 ± 0.7	4.8 ± 0.8	0.003
Total ectasia length (mm)	53.1 ± 11.1	49.3 ± 10.8	0.004

Preprocedural TIMI flow			
0/1	410 (88.9)	81 (89.0)	0.688
2	20 (4.3)	60 (6.6)	
3	31 (6.7)	4 (4.4)	

Postprocedural TIMI flow			
0/1	5 (1.1)	—	0.443
2	83 (18.0)	15 (16.5)	
3	373 (80.9)	76 (83.5)	

Markis classification			
Type I	45 (9.8)	5 (5.5)	0.001
Type II	99 (21.5)	14 (15.4)	
Type III	174 (37.7)	24 (26.4)	
Type IV	143 (31.0)	48 (52.7)	

Values are mean ± SD, *n* (%), or median (interquartile range) unless otherwise stated. CAD, coronary artery disease; CAE: coronary artery ectasia; LAD: left anterior descending artery; CX: circumflex artery; LMCA: left main coronary artery; RCA: right coronary artery; PCI/PTCA: percutaneous coronary intervention/percutaneous transluminal coronary angioplasty; SYNTAX: Synergy between PCI with Taxus and Cardiac Surgery; TIMI; thrombolysis in myocardial infarction.

**Table 5:** Univariate and multivariate logistic regression analysis for predicting CAE.

Variable	Univariate analyses		Multivariate analyses	
			Nagelkerke *R*^2^ in final step = 0.665	
	OR (95% CI)	*p* value	OR (95% CI)	*p* value
Age (years)	0.989 (0.982–0.996)	0.003	1.0121 (1.001–1.020)	0.027
Diabetes mellitus	0.389 (0.311–0.487)	<0.001	0.270 (0.191–0.382)	<0.001
Hypertension	0.646 (0.541–0.771)	<0.001	0.954 (0.738–1.233)	0.719
Dyslipidemia	0.385 (0.320–0.465)	<0.001	0.640 (0.499–0.820)	<0.001
Smoking	0.842 (0.706–1.005)	0.056		
Diagnosis (STEMI)	1.164 (1.103–1.229)	<0.001	0.983 (0.913–1.059)	0.659
FPG (mg/dL)	0.995 (0.993–0.997)	<0.001	0.998 (0.995–1.001)	0.187
Uric acid (mg/dL)	1.059 (1.003–1.117)	0.037	0.968 (0.894–1.049)	0.430
Albumin (mg/dL)	1.001 (0.967–1.037)	0.935		
Triglycerides (mg/dL)	0.999 (0.998–1.000)	0.039	1.000 (0.998–1.001)	0.439
TC (mg/dL)	0.998 (0.996–1.000)	0.027	0.998 (0.995–1.000)	0.103
HDL-C (mg/dL)	1.009 (1.005–1.013)	<0.001	1.005 (0.995–1.015)	0.305
CRP (mg/dL)	0.963 (0.931–0.997)	0.032	0.980 (0.956–1.005)	0.114
WBC (×1000/mm^3^)	0.985 (0.964–1.007)	0.188		
Basophil (×10^9^ cells/l)	1.002 (1.000–1.004)	0.085		
Hematocrit (%)	1.020 (1.012–1.028)	<0.001	1.035 (1.022–1.048)	<0.001
C-peptide (ng/dL)	2.089 (1.975–2.209)	<0.001	2.417 (2.212–2.641)	<0.001
Severity of CAD	0.682 (0.627–0.743)	<0.001	1.092 (0.956–1.248)	0.196

CAD: coronary artery disease; CAE: coronary artery ectasia; CRP: C-reactive protein; FPG: fasting plasma glucose; TC: total cholesterol; HDL-C: high-density lipoprotein cholesterol; WBC: white blood cell; STEMI: ST-elevation myocardial infarction.

**Table 6:** Univariate and multivariate cox regression analysis to evaluate the association between C-peptide, CAE, and MACE.

Variables	Univariate cox regression analyses		Multivariate cox regression analyses	
	HR (95% CI)	*p* value	HR (95% CI)	*p* value
Age	0.993 (0.986–1.000)	0.042	1.002 (0.978–1.026)	0.870
Hypertension	1.045 (0.880–1.242)	0.614		
Smoking	0.873 (0.804–0.948)	0.001	0.883 (0.645–1.1208)	0.435
Dyslipidemia	0.604 (0.503–0.727)	<0.001	0.716 (0.524–0.1008)	0.035
Diabetes mellitus	0.981 (0.900–1.070)	0.671		
Diagnosis (STEMI)	1.181 (1.122–1.242)	<0.001	0.866 (0.713–1.051)	0.146
FPG (mg/dl)	0.999 (0.998–1.000)	0.268		
Uric acid (mg/dl)	0.902 (0.855–0.952)	<0.001	1.183 (0.948–1.476)	0.138
Albumin (mg/dl)	0.761 (0.637–0.909)	0.003	0.632 (0.409–0.975)	0.038
Tyriglyceride (mg/dl)	0.999 (0.998–1.000)	0.004	1.000 (0.996–1.003)	0.849
TC (mg/dl)	0.999 (0.997–1.001)	0.268		
HDL-C (mg/dl)	1.002 (0.998–1.007)	0.321		
LDL-C (mg/dl)	0.999 (0.996–1.001)	0.247		
CRP (mg/L)	0.973 (0.946–1.001)	0.062		
Basophil, (×10^9^ cells/l)	0.996 (0.986–1.006)	0.462		
ALP (U/L)	1.000 (0.996–1.003)	0.858		
Hematocrit (%)	0.995 (0.984–1.007)	0.394		
C-peptide (ng/dl)	0.488 (0.443–0.536)	<0.001	0.213 (0.151–0.301)	<0.001
Severity of CAD	0.951 (0.878–1.031)	0.223		
HbA1c (%)	0.920 (0.882–0.959)	<0.001	1.225 (1.028–1.461)	0.024
Thrombus burden (high)	1.998 (1.693–2.358)	<0.001	0.830 (0.612–1.125)	0.229
Max. stent diameter (mm)	0.244 (0.189–0.315)	<0.001	0.819 (0.497–1.349)	0.432
Total stent length (mm)	0.897 (0.883–0.912)	<0.001	0.996 (0.949–1.045)	0.872
Preprocedural TIMI flow	1.092 (0.950–1.255)	0.215		
Postprocedural TIMI flow	1.164 (0.855–1.585)	0.335		
Culprit artery (Cx)	1.309 (1.091–1.570)	0.004	1.087 (0.856–1.382)	0.493
CAE in the culprit artery	2.601 (1.316–5.142)	0.006	2.180 (0.925–4.121)	0.358
CAE	0.332 (0.270–0.408)	<0.001	0.015 (0.004–0.0058)	<0.001

ALP: alkaline phosphatase; CAD: coronary artery disease; CAE: coronary artery ectasia; CRP: C- reactive protein; FPG: fasting plasma glucose; HR: hazard ratio; MACE: major adverse cardiovascular events; STEMI: ST-segment elevation myocardial infarction; TC: total cholesterol; HDL-C: high-density lipoprotein cholesterol; LDL-C: low-density lipoprotein cholesterol; CX: circumflex; TIMI: thrombolysis in Myocardial Infarction.

**Table 7:** Univariate and multivariate cox regression analysis to evaluate the association between DM and MACE in patients with CAE.

Variables	Univariate cox regression analyses		Multivariate cox regression analyses	
	HR (95% CI)	*p* value	HR (95% CI)	*p* value
Gender	0.801 (0.665–0.965)	0.020	0.571 (0.318–1.024)	0.060
Hypertension	0.985 (0.818–1.187)	0.875		
Diabetes mellitus	0.681 (0.396–1173)	0.166		
FPG (mg/dl)	0.985 (0.978–0.992)	<0.001	0.971 (0.959–0.183)	0.105
Uric acid (mg/dl)	0.797 (0.691–0.920)	0.002	1.069 (0.901–1.269)	0.444
Creatinin (mg/dl)	0.406 (0.137–0.881)	0.023	1.164 (0.437–3.099)	0.762
Tyriglyceride (mg/dl)	0.998 (0.995–1.000)	0.033	0.997 (0.994–1.012)	0.085
TC (mg/dl)	1.002 (0.997–1.007)	0.376		
LDL-C (mg/dl)	0.995 (0.990–1.000)	0.044	0.989 (0.981–0.1213)	0.105
GFR (ml/min)	1.003 (0.994–1.011)	0.546		
Total bilirubin (mg/dl)	0.185 (0.079–0.433)	<0.001	0.251 (0.073–0.862)	0.028
GGT (U/L)	0.976 (0.963–0.989)	<0.001	0.999 (0.984–1.014)	0.890
MPV (fL)	1.008 (0.876–1.159)	0.911		
C-peptide (ng/dl)	1.293 (1.223–1.344)	<0.001	1.254 (1.212–1.354)	<0.001

CAE: coronary artery ectasia; FPG: fasting plasma glucose; HR: hazard ratio; TC: total cholesterol; LDL-C: low-density lipoprotein cholesterol; GFR: glomerular filtration rate; GGT: gamma-glutamyl transferase; MPV: mean platelet volume.

## Data Availability

The data that support the findings of this study are available from the corresponding author upon reasonable request.
